# A Spectral Interpretable Bearing Fault Diagnosis Framework Powered by Large Language Models

**DOI:** 10.3390/s25123822

**Published:** 2025-06-19

**Authors:** Panfeng Bao, Wenjun Yi, Yue Zhu, Yufeng Shen, Haotian Peng

**Affiliations:** 1 National Key Laboratory of Transient Physics, Nanjing University of Science and Technology, Nanjing 210094, China; 223121110079@njust.edu.cn (P.B.); wjy@njust.edu.cn (W.Y.); 2 School of Aeronautical Mechanical Manufacturing, Changsha Aeronautical Vocational and Technical College, Changsha 410124, China; zhuyue19931277@163.com (Y.Z.); 15116220284@163.com (Y.S.); 3 Shenyang Institute of Automation, Chinese Academy of Sciences, Shenyang 110069, China; 4 University of Chinese Academy of Sciences, Beijing 101408, China

**Keywords:** bearing fault diagnosis, interpretable artificial intelligence, large language models, spectral feature extraction, multi-source information fusion

## Abstract

Most existing fault diagnosis methods, although capable of extracting interpretable features such as attention-weighted fault-related frequencies, remain essentially black-box models that provide only classification results without transparent reasoning or diagnostic justification, limiting users’ ability to understand and trust diagnostic outcomes. In this work, we present a novel, interpretable fault diagnosis framework that integrates spectral feature extraction with large language models (LLMs). Vibration signals are first transformed into spectral representations using Hilbert- and Fourier-based encoders to highlight key frequencies and amplitudes. A channel attention-augmented convolutional neural network provides an initial fault type prediction. Subsequently, structured information—including operating conditions, spectral features, and CNN outputs—is fed into a fine-tuned enhanced LLM, which delivers both an accurate diagnosis and a transparent reasoning process. Experiments demonstrate that our framework achieves high diagnostic performance while substantially improving interpretability, making advanced fault diagnosis accessible to non-expert users in industrial settings.

## 1. Introduction

Rotating machinery constitutes the backbone of modern industrial infrastructure, playing pivotal roles in manufacturing, energy generation, transportation, and countless other sectors [1]. Within these systems, rolling element bearings are among the most critical and ubiquitously used components [2], yet they are also highly susceptible to failures due to harsh operating conditions, fatigue, and wear [3]. Unforeseen bearing failures can lead to catastrophic equipment breakdowns, resulting in significant economic losses from unscheduled downtime, costly repairs, and potential safety hazards [4]. To address these challenges, intelligent fault diagnosis methods have attracted increasing attention, with recent works exploring early fault detection based on residual analysis in electric–hydraulic control systems [5] and AI-enhanced fault prediction under scenarios with incomplete industrial information [6].

In addition, there has been notable progress in the diagnosis of mechanical and compound faults. For example, the latest studies employ techniques such as multi-label domain adversarial reinforcement learning for unsupervised compound fault recognition [7], domain reinforcement feature adaptation with correlation alignment for compound fault diagnosis of rolling bearings [8], biologically inspired compound defect detection utilizing spiking neural networks with continuous time–frequency gradients [9], and autonomous recognition frameworks based on reinforced adversarial open set algorithms for mechanical equipment fault diagnosis [10]. Consequently, the development of robust condition monitoring and accurate fault diagnosis techniques for bearings is paramount for implementing effective predictive maintenance strategies, ensuring operational reliability, and enhancing industrial safety.

Historically, bearing fault diagnosis relied heavily on traditional signal processing methods, such as Fast Fourier Transform (FFT), wavelet analysis, empirical mode decomposition, and envelope analysis—where optimal demodulation-band selection is crucial for effective diagnostics [11,12]. Additionally, advanced techniques such as blind deconvolution [13] and cyclostationary analysis [14] have gained popularity for their effectiveness in extracting fault-related features from complex and noisy signals. While valuable, these approaches often necessitate considerable domain expertise for feature selection and interpretation and can struggle with complex signal characteristics or varying operating conditions [15]. The dawn of artificial intelligence, particularly machine learning and deep learning, has revolutionized the field [16]. Models like convolutional neural networks (CNNs), recurrent neural networks (RNNs), and autoencoders have demonstrated remarkable success in automatically extracting salient features from raw sensor data and achieving high diagnostic accuracy, often surpassing traditional methods [17].

Despite their impressive performance, a significant drawback hinders the widespread adoption and full potential of many state-of-the-art deep learning-based diagnostic systems: their inherent lack of interpretability. These models often function as “black-boxes,” taking sensor data as input and producing a diagnostic label without providing clear insight into the reasoning behind their predictions [18]. Although recent advances in semi-supervised detection, such as multi-modal imitation learning for arc detection in complex railway environments [19], unsupervised methods like collaborative adversarial domain adaptation for rotating machinery fault diagnosis [20], and physics-informed detection approaches exemplified by surrogate modeling of pantograph–catenary system interactions [21], have demonstrated strong feature extraction capabilities and robustness in challenging industrial scenarios, the interpretability of these techniques remains limited. This opacity poses considerable challenges: it erodes user trust, particularly among non-expert personnel who need to act upon the diagnostic output. Furthermore, when a model yields an incorrect diagnosis, the lack of transparency makes it exceedingly difficult to identify the cause of the error and refine the system. This interpretability gap represents a critical barrier, limiting the practical utility and reliability of advanced AI diagnostic tools in mission-critical industrial environments where accountability and understanding are essential [22].

To bridge the gap between diagnostic accuracy and interpretability in bearing fault diagnosis, this paper proposes a novel spectral interpretable framework powered by large language models (LLMs). Our approach synergistically integrates advanced signal processing, attention-augmented deep learning, and LLM-based reasoning. Specifically, vibration signals are first transformed spectrally using Hilbert- and Fourier-based encoders to highlight characteristic fault frequencies and amplitudes [23]. An attention-based convolutional neural network (CNN) then extracts robust features and provides a preliminary fault assessment. Crucially, we fuse multi-source information—including operational parameters (such as rotational speed and load), extracted spectral features, and the CNN’s initial predictions—into a structured textual representation tailored for LLM processing. This is fed into a fine-tuned LLM, adapted for the diagnostic task, which produces the final diagnosis accompanied by a transparent, step-by-step explanation of its reasoning process.

Compared with traditional approaches relying on manual feature engineering or black-box deep learning models with limited interpretability, our method offers both high classification accuracy and explicit interpretability. Leveraging the generative capabilities of LLMs, our framework delivers coherent, human-understandable explanations that directly link spectral and model-based evidence to physical fault characteristics, improving transparency over existing methods that typically provide only feature-level importance. Additionally, by fine-tuning a lightweight LLM with domain-specific data and applying resource-efficient techniques like LoRA, we facilitate practical deployment in industrial environments without depending solely on large-scale general LLM APIs. Through experiments, we demonstrate that our approach not only enhances diagnostic performance, but also makes the process understandable to non-expert users—fostering trust and paving the way for broader adoption of interpretable AI in intelligent maintenance systems.

The remainder of this paper is organized as follows: Section 2 elaborates on the proposed methodology, detailing the spectral feature extraction, CNN architecture, and LLM integration. Section 3 describes the experimental setup and presents the results. Section 4 discusses the findings and the implications of the interpretable framework. Finally, Section 5 concludes the paper and suggests future research directions.

## 2. Related Works

The field of bearing fault diagnosis has seen significant advancements, evolving from traditional signal processing techniques to sophisticated artificial intelligence-based methods. Concurrently, the demand for interpretable AI systems has grown, particularly in critical industrial applications. This section reviews pertinent research in these areas.

### 2.1. Bearing Fault Diagnosis Techniques

Traditional approaches to bearing fault diagnosis have heavily relied on signal processing techniques to extract fault signatures from vibration, acoustic, or current signals. Methods such as Fast Fourier Transform (FFT) for frequency-domain analysis [24], wavelet transforms for time–frequency analysis [25], and empirical mode decomposition (EMD) for non-stationary signal processing [26] have been widely employed. These methods often require significant domain expertise for feature selection and interpretation and can be challenged by complex operating conditions and nascent fault detection.

With the advent of machine learning, techniques like Support Vector Machines (SVMs) [27] were applied to automate the classification process based on handcrafted features. More recently, deep learning models, particularly convolutional neural networks (CNNs), have demonstrated remarkable success in automatically learning hierarchical features directly from raw or minimally processed sensor data, achieving high diagnostic accuracies [28,29]. Recurrent neural networks (RNNs) and Long Short-Term Memory (LSTM) networks have also been utilized to model temporal dependencies in vibration signals [11,30]. While these deep learning models often achieve state-of-the-art performance, they are typically regarded as “black-boxes,” offering limited insight into their decision-making processes, which hinders user trust in and adoption of safety-critical systems.

### 2.2. Interpretable Artificial Intelligence in Fault Diagnosis

The opacity of deep learning models has spurred research into eXplainable Artificial Intelligence (XAI). In the context of fault diagnosis, efforts have been made to enhance model transparency. Initial approaches often focus on post hoc explanation techniques. For instance, some methods visualize learned features or activation maps within CNNs to understand which parts of the input signal contribute most to the diagnosis [22]. Others employ model-agnostic tools like LIME [31]. Attention mechanisms have also been widely incorporated, as they inherently highlight salient input features, improving both performance and providing a degree of interpretability [32].

However, equating interpretability solely with such post hoc analysis presents a limited view. These methods, while valuable for validation, treat the model as a “black-box.” For example, researchers may verify that a model’s high attention scores align with known fault characteristic frequencies [33], or that feature magnitudes from convolutional layers strongly correlate with these physical frequencies [34]. While this confirms the model has learned a relevant pattern, it does not explain how the model derives this relationship nor embed this physical knowledge as a core component of the model’s reasoning process. Consequently, the interpretability remains superficial, making it difficult to trace the model’s logic or diagnose its failures.

A distinct and more profound approach to interpretability involves embedding physical knowledge directly into the neural network, moving towards a “physics-informed” or “knowledge-guided” paradigm. In bearing fault diagnosis, this physical knowledge is explicit: characteristic fault frequencies (e.g., BPFO, BPFI, BSF) are deterministic functions [33] of the bearing’s geometry and its operational speed. Instead of hoping a black-box model implicitly learns these relationships from data, a physics-informed model explicitly incorporates them. This approach not only renders the model’s decision-making process more transparent and trustworthy but also enhances its robustness and data efficiency, as it is constrained by established physical laws rather than relying solely on data-driven correlations.

### 2.3. Large Language Models for Explanation and Reasoning

Large Language Models (LLMs) have recently emerged as powerful tools for a wide array of natural language processing tasks, including text generation, summarization, question answering, and complex reasoning [35,36]. Their ability to comprehend context and generate human-like, coherent text presents a promising avenue for enhancing the interpretability of AI systems. While the application of LLMs in industrial diagnostics is still nascent, some preliminary studies have explored their use for tasks like generating reports from structured data or assisting in troubleshooting based on textual inputs [37]. For instance, Peng et al. [38] proposed BearLLM, which enhances bearing health management with unified signal representation, showcasing an early step towards integrating LLM capabilities in this domain.

However, directly applying general-purpose LLMs to specialized tasks like bearing fault diagnosis often requires significant prompt engineering or fine-tuning to ensure domain-specific accuracy and relevance. Furthermore, integrating numerical signal features, model predictions from other AI components (like a CNN), and operational parameters into a coherent textual explanation requires a structured approach.

## 3. Methodology

**Figure 1:**
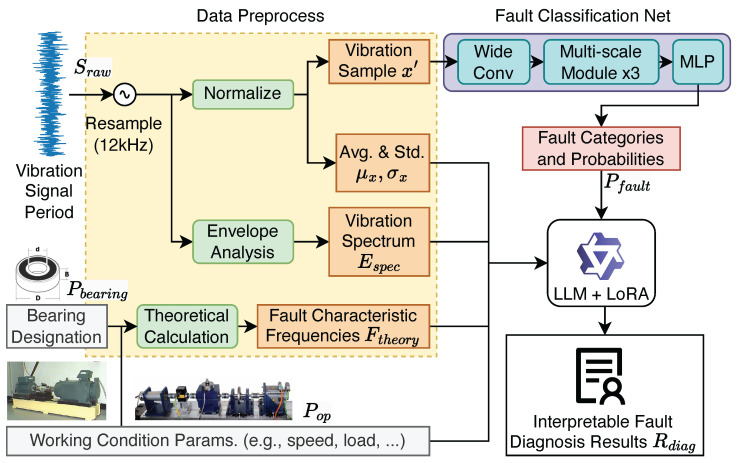
Overview of the proposed interpretable bearing fault diagnosis framework. The vibration signals and bearing information are processed to extract spectral and statistical features, which are analyzed by a multi-scale neural network and LLM to provide fault categories and interpretable diagnostic results.

Figure 1 presents the spectral interpretable bearing fault diagnosis framework proposed in this study. It consists of three main components: data preprocessing, AI-based fault classification, and fault diagnosis analysis powered by LLMs. The key distinction of our framework from conventional black-box methods is its dual-pathway analysis, which synergistically integrates a quantitative Fault Classification Network (FCN) with a qualitative, reasoning-based LLM. Unlike models that only provide a final classification label, our approach empowers the LLM to synthesize the FCN’s probabilistic output with spectral evidence and operational parameters, generating a transparent, step-by-step diagnostic report. The complete workflow, detailing each step from signal input to the final interpretable report, is summarized in Algorithm 1. The test stand images are illustrative, representing the sources of bearing and operational data, such as those from the Case Western Reserve University (CWRU) [39] bearing dataset and the Paderborn University (PU) [40] bearing dataset. This section will elaborate on the design and implementation of these modules.
**Algorithm** **1** Overall Workflow of the Spectral Interpretable Diagnosis Framework**Input:**   Raw vibration signal $S_{raw}$   Bearing geometric parameters $P_{bearing}$ (e.g., $D_{p},d_{b},n_{b},\beta$)   Operating condition parameters $P_{op}$ (e.g., rotational speed $f_{r}$, load)**Output:**   Interpretable textual diagnostic report $R_{diag}$**Part** **1:** **Data** **Preprocessing** **and** **Feature** **Extraction**1:$x\leftarrow \mathrm{SegmentAndResample}(S_{raw})$  ▹ Segment into 1 s windows and resample to 12 kHz2:$\mu_{x},\sigma_{x}\leftarrow \mathrm{CalculateStats}\left(x\right)$    ▹ Calculate mean and std dev per Equations (2) and (3)3:$x^{\prime }\leftarrow \mathrm{Normalize}\left(x,\mu_{x},\sigma_{x}\right)$  ▹ Apply z-score normalization per Equation (1) for FCN input4:$E_{spec}\leftarrow \mathrm{CalculateEnvelopeSpectrum}\left(x\right)$▹ Via Hilbert transform and FFT per Equations (4)–(6)5:$F_{theory}\leftarrow \mathrm{CalculateTheoreticalFCFs}\left(P_{bearing},P_{op}\right)$    ▹ Calculate BPFO, BPFI, BSF per Equations (7)–(9)**Part** **2:** **FCN-based** **Initial** **Classification**6:$P_{fault}\leftarrow \mathrm{FCN}\left(x^{\prime }\right)$  ▹ Feed normalized signal $x^{\prime }$ into the Fault Classification Network7:    ▹ $P_{fault}$ is the probability distribution over fault classes, per Equation (25)**Part** **3:** **LLM-based** **Interpretable** **Analysis**8:$T_{prompt}\leftarrow \mathrm{FormatPrompt}\left(P_{op},P_{bearing},\mu_{x},\sigma_{x},E_{spec},F_{theory},P_{fault}\right)$ ▹ Synthesize all information into a structured prompt for the LLM9:$R_{diag}\leftarrow {\mathrm{LLM}}_{fine-tuned}\left(T_{prompt}\right)$  ▹ Generate the final report using the fine-tuned LLM10:**return** $R_{diag}$

### 3.1. Data Preprocessing

The foundation of data-driven bearing fault diagnosis typically relies on vibration signals acquired by accelerometers. These signals exhibit characteristic changes in their frequency content when a bearing component incurs a fault. The raw vibration signals undergo a multi-stage preprocessing pipeline to prepare them for both the deep learning-based fault classification network and the large language model (LLM)-powered interpretable analysis.

**Signal** **Segmentation:** Continuous vibration signals collected online are initially segmented into non-overlapping windows, each with a duration of 1 s. This segment length, $T_{seg}$ = 1 s, is chosen for two primary reasons: Firstly, it ensures that each segment captures sufficient vibrational information for robust fault diagnosis. Secondly, this duration results in a frequency resolution of 1 Hz after performing a Fast Fourier Transform (FFT) (i.e., $\Delta f=1/T_{seg}$), which simplifies spectral analysis by ensuring that each frequency bin corresponds to an integer frequency value.

**Resampling:** Accelerometers used for data acquisition may operate at varying sampling rates. This can lead to an inconsistent number of data points per 1 s segment, which is detrimental to the performance and architecture of deep learning models that typically expect fixed-size inputs. To address this, all signal segments are resampled to a uniform sampling frequency of $f_{s}=12\;\mathrm{kHz}$. This process is often achieved by transforming the signal to the frequency domain using FFT, adjusting the spectral content to correspond to the new sampling rate, and then performing an inverse FFT (IFFT). The selection of 12 kHz represents a pragmatic trade-off between computational load and diagnostic precision; higher rates would increase computational demands, while lower rates might lead to the loss of crucial high-frequency fault signatures, thereby reducing diagnostic accuracy.

**Normalization** **for** **Deep** **Learning** **Input:** Following resampling, the amplitude of acceleration values within a segment can vary considerably depending on operating conditions and fault severity. Such variations can hinder the feature learning process of deep learning networks. Therefore, each resampled vibration signal segment, denoted as $x=[x_{1},x_{2},\ldots ,x_{N}]$, where *N* is the number of sample points in the 1-s segment at 12 kHz (i.e., N=12,000), is normalized to have a mean of 0 and a standard deviation of 1. This is achieved using standard score normalization (z-score):

(1) $$x_{i}^{\prime }=\frac{x_{i}-\mu_{x}}{\sigma_{x}}$$

where $x_{i}^{\prime }$ is the *i*-th normalized sample point, $\mu_{x}$ is the mean of the segment *x*, calculated as

(2) $$\mu_{x}=\frac{1}{N}\sum_{i=1}^{N}x_{i}$$

and $\sigma_{x}$ is the standard deviation of the segment *x*, calculated as

(3) $$\sigma_{x}=\sqrt{\frac{1}{N-1}\sum_{i=1}^{N}{\left(x_{i}-\mu_{x}\right)}^{2}}$$

These normalized segments, $x^{\prime }$, serve as the direct input to the fault classification network. The mean ($\mu_{x}$) and standard deviation ($\sigma_{x}$) calculated from each segment are also retained as descriptive statistics for the LLM-based analysis.

**Envelope** **Spectrum** **Analysis** **for** **Interpretability:** For the interpretable fault diagnosis pathway, the envelope spectrum of the vibration signal is computed. This technique is particularly effective in demodulating amplitude-modulated signals that are characteristic of bearing faults, where high-frequency resonant vibrations are modulated by lower-frequency impacts caused by defects. The process involves two main steps:

Envelope Extraction: The envelope of the resampled vibration signal x(t) is obtained using the Hilbert transform. First, the analytic signal $x_{a}\left(t\right)$ is formed:

(4) $$x_{a}\left(t\right)=x\left(t\right)+j\cdot H\left[x\left(t\right)\right]$$

where H[x(t)] is the Hilbert transform of x(t), defined as

(5) $$H\left[x\left(t\right)\right]=\frac{1}{\pi }\mathrm{P.}\mathrm{V.}\int_{-\infty }^{\infty }\frac{x(\tau )}{t-\tau }d\tau$$

(P.V. denotes the Cauchy principal value). The envelope Env(t) is then the magnitude of this analytic signal:

(6) $$Env\left(t\right)=|x_{a}\left(t\right)|=\sqrt{x{\left(t\right)}^{2}+{\left(H\left[x\left(t\right)\right]\right)}^{2}}$$

Envelope Spectrum Calculation: The FFT is then applied to this envelope Env(t), and the magnitude of the resulting spectrum is taken. This yields the envelope spectrum, which highlights the characteristic fault frequencies.

**Theoretical** **Fault** **Characteristic** **Frequency** **(FCF)** **Calculation:** To provide a basis for comparison and interpretation, theoretical Fault Characteristic Frequencies (FCFs) are calculated. These frequencies depend on the bearing’s geometric parameters and its operational speed. Key FCFs include the following:

Ball Pass Frequency Outer ring (BPFO): The frequency at which rolling elements pass a defect on the outer ring.

(7) $$f_{BPFO}=\frac{n_{b}}{2}f_{r}\left(1-\frac{d_{b}}{D_{p}}\cos\beta \right)$$

Ball Pass Frequency Inner ring (BPFI): The frequency at which rolling elements pass a defect on the inner ring.

(8) $$f_{BPFI}=\frac{n_{b}}{2}f_{r}\left(1+\frac{d_{b}}{D_{p}}\cos\beta \right)$$

Ball Spin Frequency (BSF)/Rolling Element Defect Frequency: The frequency related to a defect on a rolling element itself.

(9) $$f_{BSF}=\frac{D_{p}}{2d_{b}}f_{r}\left(1-{\left(\frac{d_{b}}{D_{p}}\cos\beta \right)}^{2}\right)$$

where $f_{r}$ is the shaft rotational frequency (e.g., in Hz). $n_{b}$ is the number of rolling elements. $d_{b}$ is the diameter of a rolling element. $D_{p}$ is the pitch circle diameter of the bearing. β is the contact angle.

These bearing designation parameters (e.g., $D_{p},d_{b},n_{b},\beta$) and working condition parameters (e.g., $f_{r}$) are used to compute these theoretical FCFs.

The computed envelope spectrum, the calculated theoretical FCFs, and the previously determined mean and standard deviation of the resampled vibration segments are then organized into a structured textual format. This compiled information serves as a comprehensive input for the subsequent LLM, enabling it to perform an integrated and interpretable fault diagnosis analysis.

### 3.2. Fault Classification Network

The normalized 1D vibration signal segments, preprocessed as described in Section 3.1, are fed into a Fault Classification Network (FCN) to obtain the probability distribution across various predefined fault categories. The architecture of the FCN is designed to effectively capture salient fault signatures from the vibration data and is detailed below. An overview of the FCN architecture is presented in Table 1.

**Wide** **Convolutional** **Layer:** The initial layer of the FCN employs a wide convolutional kernel. Specifically, a 1D convolution with a kernel size of 32, a stride of 4, and 36 output channels is used. This design choice is motivated by existing research indicating that larger receptive fields in the first convolutional layer can significantly enhance the accuracy and robustness of CNNs for fault diagnosis tasks [41]. This layer is followed by Batch Normalization (BN) and a LeakyReLU activation function. LeakyReLU is chosen over standard ReLU to mitigate the “dying ReLU” problem by allowing a small, non-zero gradient when the unit is not active, thereby preserving information and enhancing non-linearity with reduced information loss. Let $x\in R^{1\times L_{in}}$ be the input normalized vibration signal (1 channel, $L_{in}$ = 12,000 length). The output of this layer $x_{wide}\in R^{36\times L_{1}}$ is given by the following:

(10) $$x_{wide}=\mathrm{LeakyReLU}\left(\mathrm{BatchNorm}\left({\mathrm{Conv}}_{k=32,s=4}\left(x\right)\right)\right)$$

**Figure 2:**
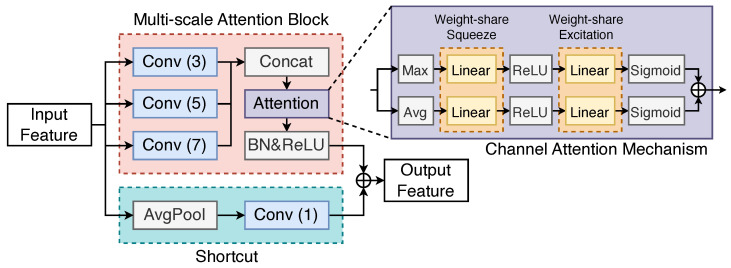
Architecture of the proposed Multi-Scale Module (MSM). The MSM integrates parallel multi-scale convolutions, a channel attention mechanism (squeeze-and-excite), and residual connections to adaptively extract and fuse discriminative features across different temporal scales.

**Multi-Scale** **Modules** **(MSM):** Following the initial wide convolution, the extracted features $x_{wide}$ are passed through a sequence of three cascaded Multi-Scale Modules (MSMs). These modules are designed to further extract discriminative fault-related features across various temporal scales. As illustrated in Figure 2, each MSM integrates multi-scale convolution, a channel attention mechanism, and residual connections.

The core operations within an MSM are as follows:

Multi-Scale Convolution: The input feature map $F_{in}\in R^{C_{in}\times L_{in}^{\prime }}$ is processed by three parallel convolutional branches with kernel sizes of 3, 5, and 7, respectively. Each branch uses a stride of 2 and appropriate padding to maintain feature map coherence. The outputs of these three branches, $F_{3},F_{5},$ and $F_{7}$, each having $C_{out}/3$ channels, are then concatenated along the channel dimension to form a combined feature map $F_{concat}\in R^{C_{out}\times L_{out}^{\prime }}$.

(11) $$\begin{array}{c} F_{3}={\mathrm{Conv}}_{k=3,s=2}\left(F_{in}\right) \end{array}$$

(12) $$\begin{array}{c} F_{5}={\mathrm{Conv}}_{k=5,s=2}\left(F_{in}\right) \end{array}$$

(13) $$\begin{array}{c} F_{7}={\mathrm{Conv}}_{k=7,s=2}\left(F_{in}\right) \end{array}$$

(14) $$\begin{array}{c} F_{concat}=\mathrm{Concat}\left(F_{3},F_{5},F_{7}\right) \end{array}$$

Channel Attention Mechanism: The concatenated multi-scale features $F_{concat}$ are then refined using a channel attention mechanism. This mechanism adaptively recalibrates the importance of each channel. It processes the input features by first computing channel-wise statistics using both global average pooling ($P_{avg}$) and global max pooling ($P_{max}$).

(15) $$\begin{array}{c} f_{avg}=P_{avg}\left(F_{concat}\right)\in R^{C_{out}} \end{array}$$

(16) $$\begin{array}{c} f_{max}=P_{max}\left(F_{concat}\right)\in R^{C_{out}} \end{array}$$

These two descriptors are then fed through a shared Multi-Layer Perceptron (MLP) to generate attention weights. The MLP consists of two fully connected (FC) layers: a dimensionality-reduction layer with ReLU activation, and a dimensionality-expansion layer with Sigmoid activation σ(·).

(17) $$w_{c}=\mathrm{MLP}\left(f_{c}\right)=\sigma \left(W_{2}\cdot \mathrm{ReLU}\left(W_{1}f_{c}\right)\right)$$

where $W_{1}$ and $W_{2}$ are the weights of the FC layers. The final channel attention weights $w_{final}\in R^{C_{out}}$ are derived by averaging the weights from the max-pooled and average-pooled paths:

(18) $$w_{final}=\left(w_{avg}+w_{max}\right)/2$$

The input feature map $F_{concat}$ is then rescaled channel-wise:

(19) $$F_{att}=F_{concat}⊙w_{final}^{\prime }$$

where $w_{final}^{\prime }$ is $w_{final}$ unsqueezed and broadcasted along the length dimension, and ⊙ denotes element-wise multiplication. This allows the network to emphasize informative features from different scales, enhancing its robustness.

Residual Connection: To facilitate training of deeper networks and prevent degradation, a residual connection [42] is incorporated. The original input to the MSM, $F_{in}$, is transformed by an average pooling layer (AvgPool with kernel size 3, stride 2) followed by a 1D convolution with a kernel size of 1 (${\mathrm{Conv}}_{k=1}$). This 1×1 convolution adjusts the channel dimensions and ensures the spatial dimensions of the shortcut path $F_{shortcut}$ match those of $F_{att}$.

(20) $$F_{shortcut}={\mathrm{Conv}}_{k=1}\left(\mathrm{AvgPool}\left(F_{in}\right)\right)$$

The output of the MSM, $F_{out}$, is then obtained by element-wise addition of the attention-processed features (after BatchNorm and ReLU) and the shortcut features:

(21) $$F_{out}=\mathrm{ReLU}\left(\mathrm{BatchNorm}\left(F_{att}\right)\right)+F_{shortcut}$$

The stacking of multiple MSMs (three in this architecture) allows the FCN to learn hierarchical features and exponentially increases the variety of receptive field scales, thereby enhancing its feature extraction capability. The residual connections within each MSM mitigate the risks associated with very deep networks, such as vanishing gradients.

**Classification** **Head:** After processing by the stack of MSMs, the resulting feature maps undergo a final feature aggregation step. Both global average pooling and global max pooling are applied across the length dimension of the output from the last MSM, $F_{MSM3}\in R^{C_{final}\times L_{final}^{\prime }}$.

(22) $$\begin{array}{c} f_{global\_avg}=P_{avg}\left(F_{MSM3}\right)\in R^{C_{final}} \end{array}$$

(23) $$\begin{array}{c} f_{global\_max}=P_{max}\left(F_{MSM3}\right)\in R^{C_{final}} \end{array}$$

These two feature vectors are then concatenated to form a comprehensive feature representation $f_{concat\_global}\in R^{2\cdot C_{final}}$.

(24) $$f_{concat\_global}=\mathrm{Concat}\left(f_{global\_avg},f_{global\_max}\right)$$

This combined feature vector is fed into a final MLP, which acts as the fault classifier. Specifically, the MLP comprises two fully connected layers: the first layer is followed by a ReLU activation function, while the second layer maps the output to the desired number of fault classes ($N_{classes}=10$). The output logits from the MLP for each class, denoted as $z_{j}$ for the *j*-th class, are then transformed using the Softmax function to obtain normalized probabilities indicating the likelihood of each fault type:

(25) $$P\left(y=j|f_{concat\_global}\right)=\frac{e^{z_{j}}}{\sum_{k=1}^{N_{classes}}e^{z_{k}}}$$

where $z_{j}$ is the logit (unnormalized score) corresponding to class *j*. The Softmax function ensures that the sum of all output probabilities equals 1, allowing the model to interpret these values as confidence scores for each possible fault category.

**Figure 3:**
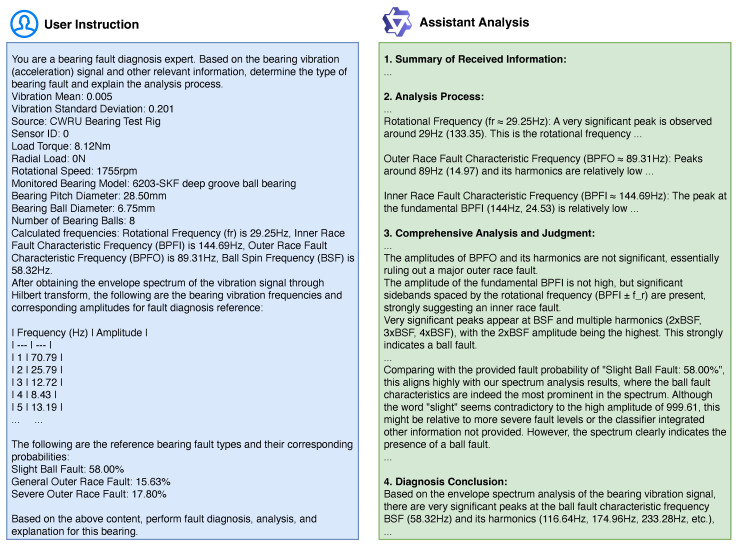
An example of interpretable bearing fault diagnosis using the fine-tuned LLM. (**Left**) The structured textual input (‘User Instruction’) compiles bearing data, operational conditions, spectral features, and FCN probabilities. (**Right**) The LLM’s textual output (‘Assistant Analysis’) delivers a step-by-step analysis, evidence-based judgment, and a conclusive diagnosis.

To facilitate interpretable fault diagnosis, the predicted class labels along with their corresponding probabilities are formatted as text and provided as input to the LLM, as illustrated in Figure 3. The LLM takes the output from the fault FCN, as well as other available information, to perform comprehensive spectral analysis and fault diagnosis. By integrating the quantitative probability distribution generated by the MLP classifier with the LLM’s reasoning and knowledge capabilities, the system enhances both the transparency and accuracy of fault diagnosis.

### 3.3. Interpretable Diagnosis with LLMs

The final stage of the proposed framework focuses on generating an interpretable diagnostic report by leveraging the capabilities of large language models (LLMs). This step aims to synthesize the information derived from the data preprocessing stage (Section 3.1) and the fault classification network (Section 3.2) into a human-readable and technically sound analysis.

**Input** **Formulation** **for** **LLM:** To enable the LLM to perform a comprehensive diagnostic analysis, all relevant information is meticulously compiled and structured into a formatted textual prompt. As illustrated in Figure 3, this input encompasses the following:

Bearing and Operational Parameters: Details such as bearing designation, geometric specifications (e.g., pitch circle diameter, ball diameter, number of rolling elements), rotational speed, and load conditions.

Signal Characteristics: Statistical properties of the vibration signal, including its mean and standard deviation.

Spectral Information: Key data points from the calculated envelope spectrum of the vibration signal, highlighting prominent frequency components and their amplitudes.

Theoretical Fault Characteristic Frequencies (FCFs): The calculated BPFO, BPFI, and BSF values relevant to the specific bearing and operational speed.

Fault Classification Network (FCN) Output: The probability distribution across different fault categories as determined by the FCN. These probabilities serve as a quantitative guide for the LLM’s diagnostic reasoning.

This comprehensive textual input provides the LLM with a detailed context of the equipment under diagnosis and the empirical evidence gathered.

**Employing Fine-Tuned Smaller LLMs:** While large-scale, cloud-based LLMs (e.g., models in the class of DeepSeek or Gemini series) demonstrate profound analytical capabilities on structured text, their application in real-time industrial settings can be constrained by factors such as network latency, data privacy concerns, API reliability, and operational costs. Given that bearing fault diagnosis is a specialized domain, a more resource-efficient approach is adopted. This study utilizes a smaller, pre-trained LLM that is subsequently fine-tuned using Low-Rank Adaptation (LoRA). This strategy aims to achieve diagnostic accuracy comparable to larger models but with significantly reduced computational overhead and enhanced deployment flexibility.

**LLM** **Fine-Tuning** **Methodology:** The development of the specialized diagnostic LLM involves a three-step fine-tuning process:

Generation of Initial Diagnostic Narratives: High-quality training data is crucial for effective fine-tuning. To generate an initial dataset of diagnostic explanations, existing bearing fault samples (each with its corresponding structured textual input as described above) are processed by advanced, general-purpose LLMs (e.g., models like DeepSeek-R1, Gemini 2.5 Pro, or similar, known for their strong reasoning and text generation capabilities). These models are prompted to produce a detailed “thought process” and a comprehensive diagnostic analysis for each sample.

Expert Review and Data Refinement: The diagnostic narratives generated by the large LLMs are then meticulously reviewed, corrected, and enhanced by human domain experts. This expert oversight ensures the factual accuracy, technical soundness, and completeness of the (prompt, response) pairs, thereby curating a high-quality dataset for fine-tuning.

Parameter-Efficient Fine-Tuning with LoRA: A pre-trained LLM of a relatively smaller scale (e.g., Qwen3-4B [43], a model from the Qwen family with approximately 4 billion parameters) is selected as the base model. This base LLM is then fine-tuned using Low-Rank Adaptation (LoRA) [44]. LoRA is a parameter-efficient fine-tuning (PEFT) [45] technique that significantly reduces the number of trainable parameters. Instead of updating all weights of the pre-trained LLM, LoRA introduces trainable low-rank matrices into specific layers of the model, typically the attention layers. If $W_{0}\in R^{d\times k}$ represents a pre-trained weight matrix, its update ΔW is approximated by the product of two smaller matrices, $B\in R^{d\times r}$ and $A\in R^{r\times k}$, such that ΔW=BA. Here, the rank *r* is much smaller than *d* and *k* (r≪min(d,k)). During fine-tuning, the original weights $W_{0}$ are kept frozen, and only the parameters of matrices *A* and *B* are updated. This approach drastically reduces computational costs and memory requirements for training and deployment, while often achieving performance comparable to full fine-tuning on specific downstream tasks.

**Interpretable** **Diagnostic** **Output:** Upon receiving the structured textual input for a new bearing sample, the fine-tuned LLM generates a comprehensive diagnostic report. As exemplified in the “Assistant Analysis” panel of Figure 3, this report typically includes the following:

Summary of Received Information: A brief reiteration of the key input parameters and observations.

Detailed Analysis Process: Step-by-step reasoning of how the LLM arrived at its conclusions. This may involve correlating the observed spectral peaks with theoretical FCFs, identifying harmonics and sidebands, and discussing the implications of specific amplitude patterns.

Comprehensive Judgment: An assessment of the bearing’s health status, integrating the evidence from the envelope spectrum, statistical features, and the FCN’s probabilistic output. The LLM may elaborate on the consistency or discrepancies between different sources of evidence.

Diagnosis Conclusion: A clear statement of the diagnosed fault type (or normal condition) and its potential severity, along with the supporting rationale.

This textual output provides maintenance personnel with not just a diagnostic label but also a clear, interpretable explanation of the fault condition, facilitating informed decision-making.

## 4. Experimental Study

### 4.1. Experimental Setup

To quantitatively evaluate the effectiveness of the proposed method, this study utilized two benchmark datasets widely adopted in bearing fault diagnosis research: the Case Western Reserve University (CWRU) [39] bearing dataset and the Paderborn University (PU) [40] bearing dataset. The specifics of each dataset are detailed as follows.

The CWRU dataset was generated from a 2 HP motor test rig. For our analysis, we used the complete set of vibration signals, collected at a sampling rate of 12 kHz or 48 kHz across all four available motor load conditions (0, 1, 2, and 3 HP). This comprehensive usage ensures a thorough evaluation under varied operational states. Faults were artificially introduced to SKF deep-groove ball bearings using electro-discharge machining (EDM) at three distinct diameters—0.007, 0.014, and 0.021 inches—to represent different severity levels. These severities were applied to three fault locations: the Inner Ring (IR), Outer Ring (OR), and Ball (B). Consequently, the nine fault categories, combined with the normal condition data across all specified loads, form the ten classes for the CWRU experiments.

The PU dataset was sourced from a modular test rig at Paderborn University, and for this work, only the data from bearings with artificial damages were selected. These damages were induced on 6203 deep-groove ball bearings using methods like drilling and EDM on the Inner Ring (IR) and Outer Ring (OR). The vibration signals were captured at a high sampling rate of 64 kHz under different operating conditions. We selected fault samples with different damage sizes on the IR and OR to represent distinct severity levels. These, combined with the healthy bearing data, were organized into the required fault classification structure.

All collected samples were randomly partitioned into training, validation, and test sets following a 7:2:1 ratio. The proposed algorithm was implemented using the PyTorch 2.7 deep learning framework. The Fault Classification Network (FCN) was trained for 50 epochs employing the cross-entropy loss function and the AdamW optimization algorithm. The initial learning rate was set to $1\times 10^{-3}$ and was subsequently halved if the validation loss did not decrease for five consecutive epochs. For the large language model (LLM) component, the pre-trained Qwen3-4B [43] model served as the base. Low-Rank Adaptation (LoRA) [44] with a rank (*r*) of 4 was integrated using the Hugging Face PEFT (Parameter-Efficient Fine-Tuning) [45] library. The LLM underwent instruction fine-tuning using a dialogue dataset curated and refined by domain experts.

### 4.2. Fault Classification Results

To validate the capability of the proposed Fault Classification Network (FCN), its performance was benchmarked against several existing models from contemporary research under identical experimental conditions. The comparative models included the following: a standard convolutional neural network (CNN), a Wide First-layer Kernel CNN (WDCNN) [41], a Multi-Scale CNN (MCNN) [11], a Transferable CNN (TCNN) [34], a Quadratic CNN (QCNN) [46], and a Stochastic CNN (SCNN) [47]. The comparative experimental results are presented in Table 2.

Table 2 systematically outlines the performance of the FCN against the selected baseline models across multiple evaluation metrics, including Accuracy, Precision, F1-Score, False Positive Rate (FPR), False Negative Rate (FNR), and Mean Squared Error (MSE). Furthermore, since all benchmarked models, including our proposed FCN, are based on convolutional operations optimized for modern computing hardware, their inference times are at the millisecond level for input sequences of 1 s. Therefore, they are all suitable for real-time or online fault diagnosis scenarios, with negligible differences in time cost for practical deployments. As evidenced by the results, the proposed FCN demonstrates superior performance across all evaluated metrics.

Specifically, the FCN achieves the highest accuracy of 0.9919, indicating its exceptional capability in correctly identifying the fault categories. In terms of precision and F1-score, which are macro-averaged across all classes, the FCN also leads with values of 0.7652 and 0.7643, respectively. This suggests a balanced performance in minimizing both false positives and false negatives for individual fault types.

Furthermore, the FCN exhibits the lowest FPR (0.0009) and FNR (0.0053), significantly outperforming other models. A low FPR implies that the FCN is less likely to incorrectly classify a normal condition as a fault, while a low FNR indicates its robustness in detecting actual faults. Correspondingly, the FCN also achieves the minimum MSE of 0.0010, reflecting the smallest deviation between the predicted probabilities and the actual labels, thereby underscoring its high classification confidence and accuracy.

In contrast, while models like SCNN and QCNN show competitive results, they do not consistently surpass the FCN in all aspects. For instance, SCNN, the second-best performing model in terms of accuracy (0.9838), has a higher FNR (0.0175) and MSE (0.0028) compared to the FCN. Similarly, other traditional CNN architectures such as the standard CNN, WDCNN, and MCNN, yield considerably lower scores across all metrics, highlighting the advancements made by more sophisticated architectures like the proposed FCN.

These results collectively affirm the effectiveness of the FCN architecture, with its specialized components such as the wide convolutional layers and multi-scale modules incorporating channel attention, in extracting discriminative features from the bearing vibration signals for accurate fault classification.

### 4.3. Analysis of Interpretability

**Figure 4:**
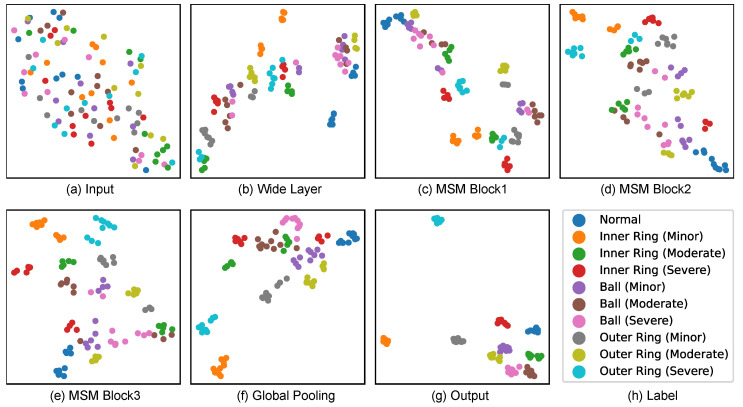
UMAP visualizations of feature representations at each layer of the FCN, illustrating the progressive separation of different fault categories from input to output.

We selected 100 samples from the CWRU dataset (10 samples from each fault category) and used Uniform Manifold Approximation and Projection (UMAP) to visualize the sample embeddings at each stage of the FCN (see Table 1), offering insight into how the network transforms raw vibration signals into separable representations for fault classification. The results are shown in Figure 4.

At the **input** stage, the normalized 1D vibration signals from different fault types are highly intermixed in the feature space. There is little to no discernible clustering by fault category, indicating the raw signals are not directly separable.

After passing through the initial **wide convolutional layer**, the samples begin to show a slight tendency to cluster. This layer, with its large receptive field, extracts broad time-domain features and provides an initial enhancement in class-related structure, but significant overlap between categories remains.

As the data traverses the cascaded **MSM blocks**, the separation between fault categories becomes increasingly distinct. Each MSM block integrates multi-scale convolution (capturing features at various temporal resolutions), channel attention (emphasizing informative features), and residual connections (facilitating deeper learning).

The **global pooling** step further aggregates the extracted features. Here, clusters corresponding to fault categories become even more compact, as both global average and max pooling distill the most salient information from each sample’s feature map, leading to refined representations that are highly class-specific.

At the **output** layer, the samples are almost perfectly separated by fault category, with minimal intra-class variance and clear inter-class boundaries. This demonstrates the FCN’s capability to transform the raw vibration signals into a latent space where different fault conditions are linearly separable.

Overall, the visualization illustrates the progressive disentanglement and clustering of fault classes through the FCN layers. The wide convolutional layer provides a strong initial feature basis, while the MSM blocks’ multi-scale and attention mechanisms enable the extraction of highly discriminative fault signatures. This hierarchical feature transformation ensures that, by the time the signals reach the classifier, the representations are highly amenable to accurate, interpretable fault diagnosis.

**Figure 5:**
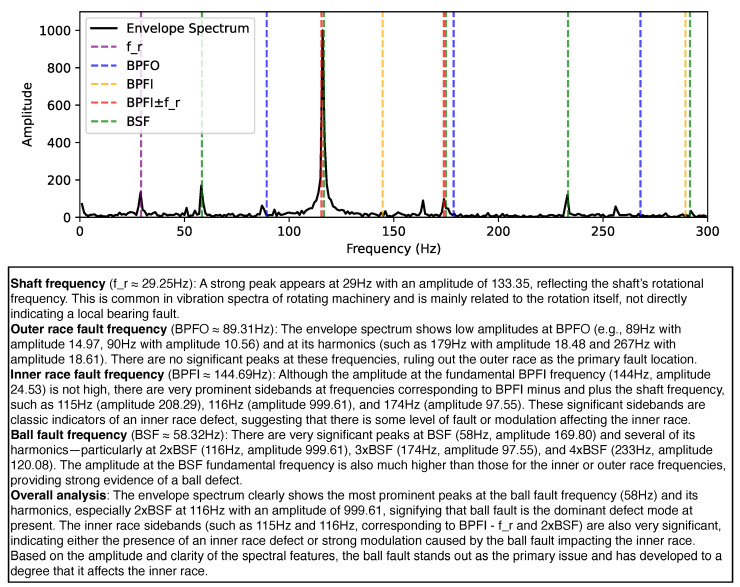
An example of spectral interpretable bearing fault diagnosis with our framework, showing (**top**) envelope spectrum annotated with characteristic fault frequencies and (**bottom**) a step-by-step diagnostic analysis generated by the LLM.

Figure 5 demonstrates an example of interpretable bearing fault diagnosis using our proposed framework. The upper part of the figure visualizes the envelope spectrum of the vibration signal, with characteristic frequencies of different fault modes (e.g., shaft frequency, BPFO, BPFI, BSF) marked as vertical dashed lines. The lower part presents a step-by-step diagnostic report generated by the fine-tuned language model.

Our approach achieves spectral interpretable diagnosis by integrating both structured numerical evidence and explicit spectral features, resulting in human-readable and logically sound diagnostic reasoning. The diagnostic process begins with structured input: the fine-tuned LLM receives a comprehensive set of information including the bearing’s geometric parameters, operational conditions, statistical properties of the acquired signal, computed fault characteristic frequencies (BPFO, BPFI, BSF), the most prominent peaks and harmonics observed in the actual envelope spectrum, and the class probabilities inferred by FCN. This amalgamation of information ensures that the language model has a holistic view of both the theoretical and empirical aspects of the diagnosis task.

In its diagnostic output, the fine-tuned LLM employs stepwise spectral reasoning, explicitly referencing the observed spectrum (as visualized in supporting figures) and systematically matching identified spectral peaks and sidebands with calculated theoretical fault characteristic frequencies. For instance, the language model discusses the presence and amplitude of peaks at BPFO and BPFI, evaluates the significance of observed harmonics or sidebands, and interprets these spectral attributes to infer potential fault locations such as the inner ring, outer ring, or ball defects. This detailed analysis enables the model to provide a transparent and data-grounded rationale for each possible fault type.

A key facet of this approach is evidence correlation. The language model aggregates diverse sources of evidence—including the strength and harmonic content of spectral peaks, the structure of sidebands, and the probabilities output by the FCN—to draw logical and substantiated inferences about fault types and their severities. The resulting analysis is explicit about why certain faults are confirmed or excluded, consistently referencing both the presence and absence of expected spectral signatures. This multi-evidence reasoning process increases the reliability and transparency of the diagnostic outcome.

Unlike traditional interpretable fault diagnosis methods—which typically focus on feature extraction or selection, such as identifying relevant frequencies, statistical parameters, or using attention mechanisms—our framework offers explicit interpretability at the output level. While conventional methods may highlight important features, they rarely provide a clear, step-by-step rationale that is directly linked to observed data in a human-understandable way. In contrast, our fine-tuned LLM generates diagnostic reports that systematically summarize all relevant input evidence, analyze each plausible fault frequency and associated spectral phenomena, and clearly justify the diagnostic decision with direct reference to the observed spectrum. These explanations, delivered in natural language, are easily understood and trusted by non-expert users.

In conclusion, our framework bridges the gap between accurate, data-driven diagnosis and practical interpretability. By integrating deep learning-based fault classification and advanced language model reasoning, this approach enables maintenance personnel to gain not only a fault label, but also a deep understanding of the underlying spectral evidence that supports the diagnosis—providing a level of explicitness and comprehensiveness that conventional methods cannot match.

### 4.4. Evaluation of Diagnostic Interpretability and Reasoning

To assess the interpretability and the quality of the diagnostic reasoning provided by the proposed framework, a blind review was conducted involving domain experts. These experts evaluated the outputs from various methods across multiple qualitative and quantitative dimensions. The compared approaches and their configurations are detailed below:

- **FCN+Mapping:** This baseline method utilizes the fault classification probabilities from the FCN, which are then directly mapped to predefined textual labels as the diagnostic output. This represents a common approach in automated systems where classification results are translated into simple reports without elaborate reasoning.
- **Ours** **(w/o** **FCN):** An ablation of our proposed method where the FCN module is omitted. The fine-tuned LLM directly analyzes the input spectral data to generate a diagnosis. This configuration is designed to evaluate the LLM’s standalone analytical capability on spectral features without the FCN’s initial probabilistic guidance, which, as hypothesized, might lead to lower diagnostic accuracy due to the absence of focused fault categorization.
- **Ours** **(w/o** **LoRA):** Another ablation of our method where the LLM (Qwen3-4B) is used without the LoRA fine-tuning. In this setup, the LLM processes both the raw spectral information and the FCN’s output probabilities to generate a diagnosis. This configuration aims to highlight the impact of domain-specific fine-tuning on the quality and relevance of the LLM’s generated explanations.
- **ChatGPT** **(GPT-4** **class** **API-based** **LLM)** [36]: Representing advanced, general-purpose large language models accessible via API. While capable of high-quality text generation and complex understanding, its application here is without specific fine-tuning for the bearing fault diagnosis task. Potential drawbacks for industrial deployment include inference costs, network latency and reliability, and data privacy concerns associated with API usage.
- **DeepSeek-R1** **(Advanced** **Reasoning** **LLM)** [48]: Representing a state-of-the-art LLM known for strong reasoning capabilities, also accessed as a general-purpose model without specific fine-tuning for this task. It is generally expected to provide higher-quality reasoning but may have significantly higher inference times and shares similar practical concerns with other API-based models for real-time industrial applications.
- **Ours** **(Full** **Method):** The complete proposed framework, integrating the FCN for initial fault probability estimation and the LoRA fine-tuned Qwen3-4B LLM for generating an interpretable diagnostic report based on all available information.

The expert evaluations, summarized in Table 3, focused on several key aspects: inference time, fault classification accuracy, understandability of the report, logical coherence of the diagnosis, relevance of the cited evidence, practical utility, and overall trustworthiness.

The results presented in Table 3 reveal several key insights into the interpretability and practical applicability of the different diagnostic approaches. The FCN+Mapping method, while achieving high accuracy (4.9) and being extremely fast (0.1s), scored very poorly on all qualitative aspects of interpretability, such as Understandability (1.2), Diagnosis Logic (1.0), and Evidence Relevance (1.1). This is expected, as it provides only a label without any underlying reasoning, underscoring the need for more advanced interpretable methods.

The ablation study highlights the importance of each component in our proposed framework. The Ours (w/o FCN) configuration, relying solely on the fine-tuned LLM to interpret spectral data, showed a significant drop in expert-perceived Accuracy (3.2). While its interpretability scores (e.g., Understandability 3.8, Diagnosis Logic 3.1) were better than FCN + Mapping, the lack of FCN’s focused probabilistic input clearly hampered the LLM’s ability to consistently arrive at the correct diagnosis. This demonstrates the synergistic value of the FCN providing a strong initial hypothesis for the LLM.

Conversely, the Ours (w/o LoRA) setup, which used the FCN probabilities but with a non-fine-tuned LLM, achieved good Accuracy (4.8). However, its scores for Understandability (3.5), Diagnosis Logic (3.9), and Evidence Relevance (3.7) were notably lower than our full method and even the general-purpose large LLMs. This indicates that while a general LLM can process the structured input, domain-specific fine-tuning via LoRA is crucial for generating highly coherent, relevant, and trustworthy diagnostic explanations tailored to the nuances of bearing fault analysis.

Comparing our full method with advanced general-purpose LLMs, ChatGPT (GPT-4 class) and DeepSeek-R1 both demonstrated high expert-rated accuracy and strong performance on qualitative metrics. DeepSeek-R1, in particular, excelled in Diagnosis Logic (5.0) and Evidence Relevance (4.9). However, these models exhibited significantly longer inference times (8.2s for ChatGPT, 28.2s for DeepSeek-R1) and considerable variability (large std dev). Beyond performance metrics, our approach offers significant advantages in deployment costs and data security, as it is designed for on-premise deployment, eliminating the need for costly API calls and mitigating risks associated with network latency and data privacy concerns inherent in general-purpose LLMs.

The Ours (Full Method) achieved an excellent balance of high expert-rated Accuracy (4.9) and outstanding scores across all interpretability dimensions: Understandability (4.9), Diagnosis Logic (4.9), Evidence Relevance (4.9), Utility (5.0), and Trustworthiness (5.0). Crucially, it accomplished this with a significantly faster and more consistent inference time (3.0 s ± 0.5) compared to the general-purpose API-based LLMs. This makes it highly suitable for practical applications where both accurate and interpretable diagnostics and timely responses are critical. The top scores in Utility and Trustworthiness particularly emphasize the practical value and reliability perceived by the experts for our proposed system. The overall rating of 4.9 for our method underscores its superiority when considering both performance and practical deployment characteristics.

This comprehensive expert evaluation strongly supports the assertion that our proposed framework, by synergistically combining a robust FCN with a domain-specifically fine-tuned LLM, provides not only highly accurate but also exceptionally interpretable, logical, and trustworthy bearing fault diagnoses, outperforming ablated versions and offering significant practical advantages over general-purpose large language models for this specialized task.

## 5. Conclusions and Future Work

This paper introduced a novel spectral interpretable bearing fault diagnosis framework that synergistically integrates advanced signal processing, a deep learning-based Fault Classification Network (FCN), and a fine-tuned large language model (LLM) to address the critical challenge of limited interpretability in conventional diagnostic systems. By transforming vibration signals into rich spectral representations and leveraging an attention-augmented FCN for accurate initial fault categorization, our framework provides a solid foundation for subsequent analysis. The core innovation lies in the utilization of a domain-specifically fine-tuned LLM, which processes structured information—comprising operational parameters, spectral features, and FCN outputs—to generate not only precise diagnostic conclusions but also transparent, step-by-step reasoning in natural language.

Experimental results obtained from benchmark datasets (CWRU and PU) demonstrated the superior diagnostic accuracy of our proposed FCN compared to several existing methods. More importantly, a comprehensive expert blind review highlighted the exceptional interpretability of our full framework. It significantly outperformed baseline methods and ablated versions in terms of understandability, diagnostic logic, evidence relevance, utility, and trustworthiness, while maintaining high diagnostic accuracy. The ablation studies confirmed the crucial roles of both the FCN’s guidance and the LLM’s domain-specific fine-tuning (via LoRA) in achieving these results. Our approach provides a more practical and deployable solution with faster inference times compared to general-purpose large LLMs, making it well-suited for industrial applications. By bridging the gap between high performance and clear interpretability, this work offers a significant step towards more transparent, reliable, and user-centric intelligent fault diagnosis systems.

While the proposed framework has demonstrated considerable promise, several avenues for future research and development can be explored: **Generalization to Diverse Machinery and Faults:** Future efforts will focus on extending the framework’s applicability to other types of rotating machinery (e.g., gears, motors, pumps) and a broader range of fault types and severities. **Multi-Modal Data Fusion:** Incorporating data from additional sensor modalities (e.g., acoustic emissions, temperature, motor current signature analysis) into the LLM’s input could provide a more holistic view of machine health and further enhance diagnostic accuracy and the richness of explanations. **Quantitative Evaluation of Interpretability:** Developing more objective and standardized quantitative metrics for evaluating the quality, completeness, and factual correctness of the generated explanations remains an important research direction, moving beyond subjective expert ratings. **Task-specific LLM Adaptation**: The development or fine-tuning of LLMs specifically tailored for fault diagnosis tasks. This may involve incorporating domain-specific knowledge during pre-training, designing task-oriented prompt strategies, or developing lightweight, efficient LLM architectures suitable for industrial deployment. By pursuing these future directions, we aim to further enhance the capabilities and practical utility of interpretable AI in predictive maintenance and intelligent manufacturing.

## Figures and Tables

**Table 1:** Architecture of the Fault Classification Network (FCN).

Layer Name	Input Channels	Output Channels	Kernel Size/Stride/Padding	Activation	Other Operations
Input	1	-	-	-	Normalized (1 × 12,000)
Wide Layer	1	36	32/4/15	LeakyReLU	BatchNorm1D
MSM Block 1	36	108	Multi-Scale (3,5,7)/2, Shortcut (1)/1	ReLU	Attention, BatchNorm, Residual
MSM Block 2	108	216	Multi-Scale (3,5,7)/2, Shortcut (1)/1	ReLU	Attention, BatchNorm, Residual
MSM Block 3	216	216	Multi-Scale (3,5,7)/2, Shortcut (1)/1	ReLU	Attention, BatchNorm, Residual
Global Pooling	216	432	-	-	AdaAvgPool, AdaMaxPool, Concatenate
FC Layer 1	432	128	-	ReLU	Linear
FC Layer 2 (Output)	128	10	-	Softmax	Linear

Note: For MSM blocks, “Multi-Scale” refers to the parallel convolutional branches with kernel sizes 3, 5, and 7, all with stride 2 and appropriate padding. “Shortcut” refers to the Conv1D in the residual path after an AvgPool.

**Table 2:** Performance comparison of different models for fault diagnosis.

Model	Accuracy	Precision	F1-Score	FPR	FNR	MSE
CNN	0.8653	0.6359	0.6325	0.0139	0.1347	0.0178
WDCNN	0.8565	0.5971	0.5866	0.0161	0.1738	0.0178
MCNN	0.8634	0.6540	0.6431	0.0165	0.1387	0.0216
TCNN	0.9317	0.7117	0.7051	0.0080	0.0756	0.0097
QCNN	0.9491	0.7000	0.6974	0.0058	0.0949	0.0068
SCNN	0.9838	0.7651	0.7637	0.0019	0.0175	0.0028
**FCN**	**0.9919**	**0.7652**	**0.7643**	**0.0009**	**0.0053**	**0.0010**

Note: FPR = False Positive Rate; FNR = False Negative Rate; MSE = Mean Squared Error. Precision and F1-score are calculated for each class and then averaged using the macro-average method. Bold values indicate the best performance among all models.

**Table 3:** Expert evaluation of interpretability and diagnostic quality for different methods.

Method	Time (s) ± Std	Accuracy	Understand	Diagnosis Logic	Evidence Relevance	Utility	Trustworth	Overall Rating
**FCN + Mapping**	0.1 ± 0.0	4.9	1.2	1.0	1.1	1.3	1.4	1.6
**w/o FCN**	2.9 ± 0.5	3.2	3.8	3.1	3.3	3.5	3.4	3.1
**w/o LoRA**	2.9 ± 0.5	4.8	3.5	3.9	3.7	4.0	4.1	4.0
**ChatGPT 4.1**	8.2 ± 4.3	4.9	4.8	4.7	4.5	4.6	4.7	4.5
**Deepseek R1**	28.2 ± 11.2	4.9	4.9	5.0	4.9	4.8	4.9	4.1
**Ours**	3.0 ± 0.5	4.9	4.9	4.9	4.9	5.0	5.0	4.9

Note: Expert ratings are on a scale of 1 (worst) to 5 (best). Time indicates the average inference time per sample with standard deviation. The qualitative evaluation metrics are defined as follows: **Accuracy**: Expert-perceived correctness of the final diagnosis. **Understand**: Clarity and comprehensibility of the generated explanation. **Diagnosis Logic**: Logical coherence and soundness of the reasoning process. **Evidence Relevance**: Relevance of the cited evidence to the diagnostic conclusion. **Utility**: Practical value and applicability of the diagnostic report for real-world tasks. **Trustworth**: The expert’s level of trust in the model’s conclusion and explanation. **Overall Rating**: A holistic evaluation considering all performance and qualitative factors.

## Data Availability

Data are contained within the article.
